# Validation of an Automated Cardiothoracic Ratio Calculation for Hemodialysis Patients

**DOI:** 10.3390/diagnostics13081376

**Published:** 2023-04-09

**Authors:** Hsin-Hsu Chou, Jin-Yi Lin, Guan-Ting Shen, Chih-Yuan Huang

**Affiliations:** 1 Department of Pediatrics, Ditmanson Medical Foundation Chia-Yi Christian Hospital, Chiayi 600566, Taiwan; 2 Department of Bioinformatics and Medical Engineering, Asia University, Taichung 413305, Taiwan; 3 Innovation and Incubation Center, Ditmanson Medical Foundation Chia-Yi Christian Hospital, Chiayi 600566, Taiwan; 4 Division of Nephrology, Department of Internal Medicine, Ditmanson Medical Foundation Chia-Yi Christian Hospital, Chiayi 600566, Taiwan; 5 Department of Sport Management, College of Recreation and Health Management, Chia Nan University of Pharmacy and Science, Tainan 717301, Taiwan

**Keywords:** cardiothoracic ratio (CTR), U-Net, deep learning, hemodialysis, chest X-ray

## Abstract

Cardiomegaly is associated with poor clinical outcomes and is assessed by routine monitoring of the cardiothoracic ratio (CTR) from chest X-rays (CXRs). Judgment of the margins of the heart and lungs is subjective and may vary between different operators. Methods: Patients aged > 19 years in our hemodialysis unit from March 2021 to October 2021 were enrolled. The borders of the lungs and heart on CXRs were labeled by two nephrologists as the ground truth (nephrologist-defined mask). We implemented AlbuNet-34, a U-Net variant, to predict the heart and lung margins from CXR images and to automatically calculate the CTRs. Results: The coefficient of determination (R^2^) obtained using the neural network model was 0.96, compared with an R^2^ of 0.90 obtained by nurse practitioners. The mean difference between the CTRs calculated by the nurse practitioners and senior nephrologists was 1.52 ± 1.46%, and that between the neural network model and the nephrologists was 0.83 ± 0.87% (*p* < 0.001). The mean CTR calculation duration was 85 s using the manual method and less than 2 s using the automated method (*p* < 0.001). Conclusions: Our study confirmed the validity of automated CTR calculations. By achieving high accuracy and saving time, our model can be implemented in clinical practice.

## 1. Introduction

The cardiothoracic ratio (CTR), first mentioned in 1919 [1], is a commonly used parameter for evaluating cardiomegaly, which is diagnosed if CTR > 50% [2,3]. It has good sensitivity (86.2%) and negative predictive value (74.0%) for identifying left ventricular dilation [4]. There are several different ways to calculate the CTR, the most common of which is the ratio of the length of the greatest horizontal heart span divided by the length of the greatest horizontal lung cavity span.

The CTR is related to the clinical outcomes of patients undergoing hemodialysis, including cardiovascular events and all-cause mortality [5,6,7]. This parameter is easily calculated from chest X-rays (CXRs), and serial monitoring of the CTR may have clinical benefits for patients undergoing hemodialysis. Routine CXR examination and calculation of the CTR for all hemodialysis patients are also requested by the Taiwan Nephrology Association. In our hemodialysis unit, all patients receive a routine CXR evaluation after hemodialysis at least twice a year, usually during April and October. Manual calculation of the CTR by residents or nurse practitioners is required for all CXR images in our clinical hemodialysis room practice. However, calculating all these CTRs is not only time consuming; it also exhausts manpower. Furthermore, judgment of the margins of the heart and lungs is subjective and may vary between different operators, which may result in discrepancies in the CTR calculated from the same CXR. Misinterpretation of the CTR may lead to inappropriate dialysis prescriptions and cause complications in hemodialysis patients. A false-positive CTR of greater than 50% may lead to dialysis prescriptions with higher ultrafiltration volumes, which have been associated with intradialytic hypotensive episodes [8]. In contrast, a false-negative CTR of greater than 50% may lead to dialysis prescriptions with insufficient water removal, causing volume overload, which is responsible for hypertension in the end-stage renal disease (ESRD) population [9]. Long-term volume overload is considered a major underlying risk factor for all-cause and cardiovascular death in ESRD patients [10]. Therefore, we aimed to establish an automated cardiothoracic ratio calculation system to save time and labor for our staff and improve the accuracy of the calculation.

With the assistance of powerful computer hardware today, medical images can be processed by deep learning [11], and the heart and lung fields can be accurately segmented and marked. Then, the CTR can be calculated accordingly by traditional methods. Similar methods have been shown to be accurate and effective [12,13,14,15,16] in previous works. However, hemodialysis patients are predisposed to a considerable prevalence of pulmonary complications (e.g., pulmonary edema 8.20–20.50%, pleural effusions 22.95–33.80%) [17,18]. The incidence of permanent pacemaker implantation was 5.93- and 3.50-fold greater in hemodialysis and peritoneal dialysis patients than in controls (1.44 and 0.85 versus 0.24 per 1000 person-years, respectively) [19]. In an Irish study, the overall prevalence of central venous catheter use was 54% among hemodialysis patients. These factors can make automated segmentation tasks more challenging [20]. The aim of this study was to use deep-learning algorithms to establish an automated CTR calculation system for hemodialysis patients.

## 2. Materials and Methods

This study was approved by the Institutional Review Board of Ditmanson Medical Foundation Chia-Yi Christian Hospital (IRB2022082), which waived the need for written informed consent due to the retrospective nature of the study.

### 2.1. Image Dataset

Four hundred thirteen patients receiving regular hemodialysis aged >19 years in our hemodialysis unit from March 2021 to October 2021 were enrolled. CXR posterior-anterior (PA) views were acquired from the Picture Archiving Communication System (PACS) of our radiology department. Digital Imaging and Communication in Medicine (DICOM) images were converted into grayscale JPEG format in their original resolution, which ranged from 2571~3320 by 2800~3408. Most patients underwent routine CXR examinations after hemodialysis in both April and October 2021. CXR images were excluded if they were not taken immediately after hemodialysis, or if the image was taken during hospitalization. Masks of the lungs and heart in the CXRs were manually labeled by a board-certified nephrologist and were reviewed and verified by another senior board-certified nephrologist. Consensus was achieved by discussion between the two nephrologists if disagreement arose in the segmentation mask creation or CTR measurement. In addition, we obtained the CTRs of these chest X-rays as determined by clinical staff (i.e., nurse practitioners), who manually calculated the ratio of the maximum transverse cardiac dimension to the maximum transverse internal thoracic cavity dimension.

The training and validation datasets were composed of all CXR images taken in April 2021 in our hospital. The testing dataset was composed of the CXR images taken of hemodialysis patients during October 2021 in our hospital to evaluate the diagnostic performance and reliability of our image segmentation model. The datasets are representative of the clinical setting in our hemodialysis unit. To prevent overfitting and improve the diversity and generalizability of the model for lung and heart segmentation, a publicly available image dataset from the Japanese Society of Radiological Technology (JSRT) [21] was employed. This dataset contains 247 PA CXRs, of which 154 had lung nodules, and 93 had no lung nodules. All CXRs had a resolution of 2048 × 2048 pixels. One hundred forty-seven CXRs were randomly selected for our training dataset.

### 2.2. Neural Network/Deep-Learning Model for Semantic Segmentation

**Figure 1:**
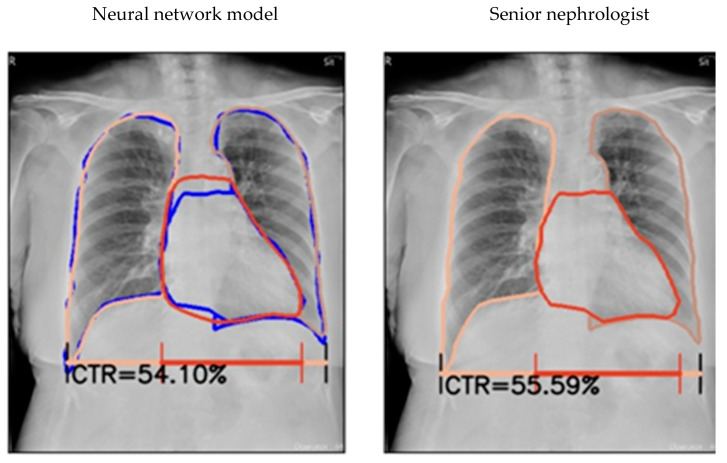
Illustration of the segmentation of the heart and lungs margin by manual annotation and the neural network model. The blue line was predicted by the neural network model, and the red and orange lines are the cardiac and lung margins, respectively, annotated by a senior nephrologist.

U-Net has been widely applied in the segmentation of medical images and can be used to extract location information with high accuracy [22]. It is known for its ability to handle small, noisy, and highly imbalanced datasets and for its robust performance on a wide range of image segmentation tasks. This model was developed from an autoencoder and consists of a contracting path (also known as the encoder) and an expanding path (also known as the decoder). The encoder and decoder are connected by a series of skip connections, which helps to preserve spatial information and improve the performance of the network. Each output character can be matched with the decoder layer to reconstruct the original images and predict a high-resolution mask output. This technique can reduce noise signals and improve segmentation accuracy. U-Net can be adapted with various types of encoders; here, we implemented AlbuNet-34, a variant of the U-Net architecture that deploys ResNet as an encoder, to predict the heart and lung margins from CXR images. After the features of the input image are obtained through ResNet34, to increase the receptive field, the output of stage 5 is downsampled through a max pooling layer. During the entire encoder process, the model mainly performs downsampling 6 times. At this time, the resolution of the feature map is 1/64 of the original input image. Therefore, it is necessary to perform upsampling 6 times using decoder blocks to restore the resolution of the image. At the end of the model, the output goes through a 3 × 3 convolutional layer, where the number of filters is the number of categories. The connection method used in AlbuNet-34 between the encoder and decoder is the same as in U-Net. AlbuNet-34 has been shown to outperform custom pretrained encoders and other commonly used variants of the U-Net architecture [23]. Then, to determine the cardiothoacic ratio (CTR), we utilized a computer program to calculate the ratio of the maximum transverse cardiac dimension to the maximum transverse internal thoracic cavity dimension, as indicated by the applied masks. An example of the determination of the CTR after heart and lung segmentation is shown in Figure 1.

### 2.3. Experimental Settings

We implemented the PyTorch framework (https://www.pytorch.org, accessed on 1 November 2021) with a CUDA backend to run our method and trained the entire network using a stochastic gradient descent (SGD) optimizer (learning rate 0.0001, momentum = 0.99) using an NVIDA RTX 3090 GPU. The loss function was the sum of the Jaccard loss. To improve the generalizability and accuracy of our deep-learning model, it was pretrained with ImageNet [24] and fine-tuned with our training dataset with both heart and lung margin annotations. ImageNet is a large dataset of labeled images that is commonly used for training and evaluating image classification models in deep learning. It contains over 14 million images, spanning more than 1000 different object categories. It has been used to train models for a wide range of applications, including object detection, image segmentation, and video analysis. The initial weights of ResNet34 in AlbuNet-34 were trained on the ImageNet dataset instead of random weights in the pretrained algorithms to facilitate fine-tuning our model to obtain better generalizability. All images were resized to 512 × 512. In the process of training, we used various methods for data augmentation, including RandomResizedCrop, ShiftScaleRotate, RandomBrightnessContrast, InverImg, ElasticTransform, GridDistortion, and OpticalDistorsion, to increase the diversity of the training dataset.

### 2.4. Statistical Analysis

Descriptive statistics include the mean and standard deviation (SD) for continuous variables and the proportion for categorical variables. To evaluate the similarity between the neural network model-predicted mask output and the nephrologist-defined (ground-truth) margins of the heart and lungs, the mean intersection over union (mIoU) and average Dice coefficient (ADC) were used as performance metrics. The Dice coefficient was calculated using the following formula: 2 × PPV × TPR/(PPV + TPR), where PPV and TPR denote the positive predicted value and true positive rate, respectively, and ADC is obtained by taking the average of the Dice coefficient across all classes. The formula for the IoU is as follows: TP/(TP + FP + FN), where TP, FP, and FN indicate the number of pixels for true positives, false positives, and false negatives, respectively, and mIOU is calculated by taking the average of the IOU values across all classes. The coefficient of determination (R^2^) (linear regression of CTR by neural network on CTR by nephrologist) and relative changes (difference between CTR by neural network and CTR by nephrologist) were used to examine the similarity of the CTR calculated between the prediction neural network model and the nephrologist-defined mask. Cardiomegaly was considered for a CTR value greater than 50% in accordance with regular clinical practice. The diagnostic performance on ground-truth CTRs > 50% was evaluated in terms of accuracy, sensitivity, specificity, and area under the receiver operating characteristic (ROC) curve (AUC). A t-test was used to assess whether there was a statistically significant difference between the CTR obtained by the neural network model and that calculated by nurse practitioners. A *p* value less than 0.05 was considered significant.

## 3. Results

### 3.1. Patient Characteristics

The included patients were divided into a group with a CTR (obtained by the nephrologists) greater than 50% and another group with a CTR less than or equal to 50%. The baseline characteristics of the patients are shown in Table 1. Patients with a CTR greater than 50% were older, were more likely to be female, had a lower post-hemodialysis body weight, and had more left ventricular hypertrophy (LVH).

In total, there were 460 CXR images in the training dataset (68% from our hemodialysis patients taken in April 2021 and 32% from the JSRT dataset), 54 CXR images in the validation dataset (all from our hemodialysis patients taken in April 2021) and 413 CXR images (taken in October 2021 from our hemodialysis patients) in the testing dataset. Four hundred thirteen patients in our hemodialysis unit were included; the mean age of the patients was 65.9 ± 12.0 years, and 56.2% were male. The mean CTR was 53.4 ± 6.1%, and 302 patients (73.1%) had a CTR value greater than 50%.

### 3.2. Performance in Image Segmentation of the Lungs and Heart

There was a high degree of overlap between the manual and automatic segmentation masks. The mean IoU (mIoU) and ADC- for segmentation of the heart and lungs in the validation dataset were 0.935 and 0.966, respectively. These metrics remained highly consistent in the testing dataset, with an mIoU and ADC of 0.950 and 0.974, respectively, in our hemodialysis patients, outperforming the CXR images from the JSRT dataset. The CTR also showed a high correlation between the automatic calculation and the nephrologist-defined mask, with R^2^ values in the validation and testing datasets of 0.950 and 0.965 (Table 2), respectively.

### 3.3. Comparison between Automatic and Manual CTR Calculation

**Figure 2:**
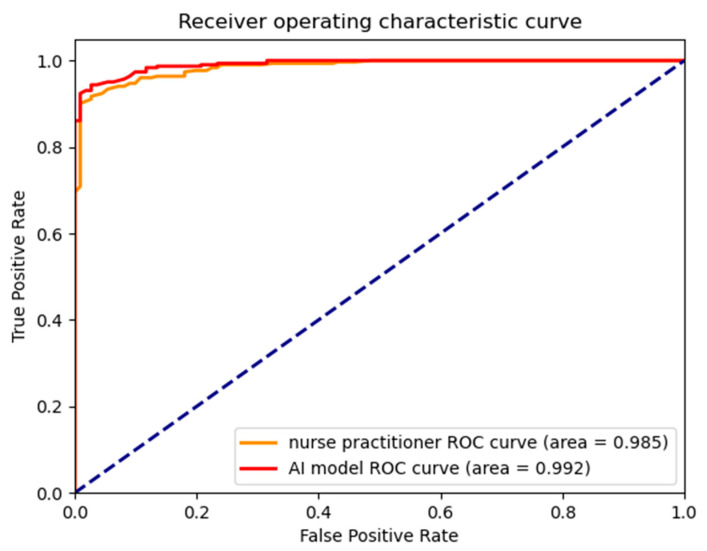
ROC curves and AUCs in the detection of cardiomegaly (CTR > 50% as calculated by the nephrologists) according to automated and manual CTR calculations.

The calculation of CTR is usually performed by nurse practitioners or resident doctors in our hemodialysis unit. We also assessed the accuracy of the CTR calculated by nurse practitioners or resident doctors (Table 3). The mean absolute difference between the CTR calculated by the nurse practitioners and the ground-truth CTR was 1.52 ± 1.46%. There were 128 (31%) CXR images with an absolute CTR difference ≥ 2% relative to the nephrologist-defined mask. In contrast, the mean absolute difference between our neural network model and the nephrologist-defined mask was 0.83 ± 0.87%, and 35 (8%) CXR images had an absolute CTR value difference ≥ 2% (all *p* < 0.001). The average time taken in calculating the CTR value by the neural network model and the clinical staff was 1 s and 85 s, respectively. The performance in detecting cardiomegaly was comparable between the automated and manual CTR calculation methods (neural network model: accuracy 94.9%, sensitivity 96.4%, specificity 91.0%, AUC 0.992; manual calculation by nurse practitioner: accuracy 92.5%, sensitivity 90.1%, specificity 99.1%, AUC 0.985, Figure 2). Scatter plots comparing the CTRs calculated by the neural network with the CTRs by the nephrologists and the CTRs calculated by the nurse practitioners with the CTRs calculated by the nephrologists are shown in Supplemental Figure S1. Bland–Altman plots comparing the neural network with the nephrologists and the nurse practitioners with the nephrologists are shown in Supplemental Figure S2. The mean duration of the manual CTR calculation was 85 s, whereas that of the automated calculation was 2 s (*p* < 0.001).

## 4. Discussion

The lung and heart segmentation methods of our study had good IoUs and Dice coefficients in both the validation and testing datasets. The automated CTRs calculated accordingly were highly correlated with those calculated by senior board-certified nephrologists and were superior to those calculated by nurse practitioners. These automated CTR calculations were more precise, robust, and less biased than the CTRs manually calculated by the nurse practitioners in routine CXR examinations for hemodialysis patients. Regarding the performance in detecting cardiomegaly, the automated CTRs slightly outperformed the nurse practitioners. These results validated the automated CTR calculation as a solid and robust method for implementation in daily clinical practice. This method not only improved accuracy in calculating the CTR but also reduced the workload of the staff of the hemodialysis unit.

To detect cardiomegaly in chest X-rays using deep learning, there are two main approaches: classification-based and segmentation-based methods. Candemar et al. employed a classification-based method to detect cardiomegaly in chest X-rays [25] by using convolutional neural networks (CNNs) to learn relevant features from the input images and classify them as either cardiomegaly or not. However, this approach only provides a binary decision, without providing information about the location or extent of the cardiomegaly region. In contrast, we employed a segmentation-based method, in which the model would be trained to first segment the region of interest (heart and lungs) from the input chest X-ray image and then identify whether the segmented region contains cardiomegaly or not. This approach can be more accurate, as it provides more detailed information about the location and extent of cardiomegaly in the image and facilitates the removal of disturbances from extrapulmonary soft tissue and bone structures. It also provides an accurate estimation of CTR, which is a crucial indicator for nephrologists in determining the need for dry weight adjustments in dialysis patients.

We conducted experiments on the publicly available JSRT dataset and compared the results with three other different methods in currently published literature (Supplemental Table S1) [26,27,28]. Our model ranked second in average Dice coefficient after Eslami et al.’s work with the pix2pix model. However, our model outperformed the pix2pix model in the image dataset from hemodialysis patient. Thus, the pix2pix model’s generalization ability is low and insufficient to handle most hemodialysis patients.

Cardiomegaly is an important predictor of various clinical outcomes in patients undergoing hemodialysis. A study of 3436 participants undergoing hemodialysis in Japan showed that a higher CTR was associated with a higher risk for both all-cause mortality and cardiovascular events [5]. Another study of 387 patients undergoing regular hemodialysis showed that CTR > 55% was an independent factor related to 2-year all-cause mortality [6]. A study with 2586 pairs of patients with normal and high CTRs matched by propensity scoring showed that a baseline CTR > 50% was associated with increased mortality and morbidity in patients with heart failure [7]. Among dialyzed patients infected with COVID-19 in Taiwan, deceased patients had a higher cardiothoracic ratio than surviving patients (0.61 vs. 0.55, *p* = 0.036) [29].

Previous studies have shown that CTRs determined by segmentation-based methods using deep learning can identify cardiomegaly with good accuracy [12,13,14,30]. The CXRs of the general population were selected for model training, validation, and testing. However, there are differences in the CXR imaging characteristics between the general population and hemodialysis patients due to the high prevalence of pulmonary disease, including atelectasis, vascular congestion, parenchymal consolidation, and parenchymal scarring-fibrosis [31], which may obscure the heart border. Furthermore, hemodialysis patients may have central venous catheters, such as Perm-Caths, or heart implants, such as pacemakers, which may make the segmentation task more challenging. Thus, we included both a public CXR image dataset and CXRs from our hemodialysis patients in the training and validation datasets to reinforce the generalizability of the neural network/deep learning model segmentation. Additionally, we included only CXRs from hemodialysis patients in the testing dataset to evaluate the diagnostic performance in hemodialysis patients. The segmentation masks were highly correlated with the nephrologist-defined masks (ground truth), with excellent mIoU and average Dice coefficient (ADC) values in both the validation and testing datasets.

There are several circumstances that could obscure the borders of the heart and lungs on CXRs. During annotation of the lung and heart borders, the most common areas with ambiguous borders are the upper and lower parts of the heart. The upper part of the heart is within the mediastinum, where no clear border exists among the heart, aorta, and superior vena cava. The lower border of the heart, meanwhile, is in contact with the diaphragm, where minor pleural effusions and vague diaphragm–heart borders make it difficult to define, even for senior nephrologists. The neural network model tends to underperform in these two areas, marking bulging or concave borders at times. Nonetheless, this poor performance did not alter the results of the automated CTR calculation. However, when there are large pleural effusions or areas of lung collapse, the neural network model also has difficulty marking the margins accurately. Thus, the feasibility of the computer-aided detection of cardiomegaly without human intervention is limited. When using CXRs with lesions obscuring the border of the heart, the performance dropped significantly without human intervention [14]. To avoid erroneous CTR calculations due to inaccurate segmentation, we designed a human-monitored automated CTR calculation system and incorporated it into our electronic health record (EHR) system. Our staff can review the segmentation results and then approve the CTRs if the result is correct; otherwise, they can manually input the correct CTR data if the segmentation area is obviously wrong.

In a study evaluating the effects of sample size on U-Net-based organ segmentation, the slope of the Dice similarity coefficient (DSC) stabilized after 200 cases and showed minimal changes as the number of cases increased further. The DSCs stabilized at a smaller sample size with the incorporation of data augmentation for all organs except the heart [32]. We applied data augmentation methods provided by PyTorch to increase the sample size and thus the accuracy after training, however, a few erroneous segmentations were still generated. In future studies, we may incorporate some newly developed data augmentation methods [33] to improve model performance in segmenting the heart.

Variations in the manually calculated CTR may result in inappropriate hemodialysis prescriptions and inadequate ultrafiltration for hemodialysis patients. In a previous study, the interobserver variation was 2.13% in the general population [3]; this variation may be higher in hemodialysis patients because of the higher prevalence of pulmonary disease or implants, which can obscure the heart or lung margins. The CTR can also be used to monitor the effect of different interventions and provide prognoses for hemodialysis patients. In patients with isolated congenital complete atrioventricular block (CCAVB), the CTRs were significantly different (*p* < 0.001) between symptomatic and asymptomatic patients after pacemaker therapy (52% and 48%, respectively) [34]. Changes in the CTR during serial monitoring of the same patient were reported to be correlated with higher mortality in a hemodialysis population [35]. We can also assess the effect of dry weight reduction on hypertension or fluid overload according to the serial monitoring of the CTR to evaluate the effects of interventions. By using automated CTR calculations, the results of our study provide more consistent data and may be used to improve clinical decision making and provide good correlations for future CTR-related studies. Our model also reduced the labor costs required by our staff in calculating the CTRs.

The strength of this study is the development of a neural network/deep-learning model for segmenting the lungs and heart with good IoUs and Dice coefficients with respect to the nephrologist-defined mask. The automated CTR calculations correlated well with the nephrologist-defined CTR and were more accurate than those of the nurse practitioners who are responsible for the routine CTR calculations in our hemodialysis unit. We used various data augmentation methods to increase the size of the training dataset to obtain better prediction results. In addition, CXRs from different hemodialysis units may have different density characteristics due to differences in the X-ray machine settings. To avoid neural network model overfitting, we included a publicly available JSRT image dataset in the training dataset to increase the robustness and generalizability of the neural network model. However, one weakness of this study is that the model still calculated the CTR erroneously at times due to the poor segmentation of the lungs and heart. Some of these errors were due to the presence of implants, and some were due to vague heart borders. However, even the senior nephrologists found it difficult at times to define the lung and heart margins. This is one of the limitations of CTR detection with plain CXRs [36], and chest computed tomography or magnetic resonance imaging [13] may be needed to obtain an accurate CTR when the above circumstances are present. To avoid erroneous segmentation with inaccurate CTR data, we developed an option for manual input to override any poorly predicted automated CTRs.

The segmentation of the lung and heart fields represents a foundational step for the subsequent evaluation of lung and heart diseases. The cardiothoracic ratio is the first clinical application that we have implemented in our clinical practice. Our goal is to evaluate lung edema, pleural effusion, and other clinical markers to determine whether a neural network can generate better surrogates with chest X-rays, enabling the detection of a wide range of clinical conditions and diseases.

## 5. Conclusions

Our study confirmed the validity of automatically calculated CTRs, which were slightly better than those calculated by the hemodialysis staff and provided high accuracy and reliability compared to nephrologist calculations. A neural network-assisted CTR calculation system can improve the quality of routine CTR evaluation and reduce the time and labor costs of our staff in routine practice.

## Figures and Tables

**Table 1:** Basic characteristics of all patients, patients with a CTR (obtained by the nephrologists) ≤ 50%, patients with a CTR >50%, and *p* values between patients with CTR ≤ 50% and with CTR > 50%.

	All Patients	CTR ≤ 50%	CTR > 50%	*p* Value
Patient number	413	112	301	
Age (±SD)	65.0 ± 11.9	57.9 ± 11.2	67.6 ± 11.1	<0.001
Sex, male (*n*, %)	236 (57.1%)	82 (73.2%)	154 (51.2%)	<0.001
Dialysis vintage, years (±SD)	7.43 ± 6.66	7.26 ± 6.61	7.87 ± 6.81	0.414
Comorbidities				
History of myocardial infarction (*n*, %)	78 (18.9%)	17 (15.2%)	61 (20.3%)	0.24
LVH	264 (63.9%)	50 (44.6%)	214 (71.1%)	<0.001
CAD	199 (48.2%)	45 (41.1%)	153 (50.8%)	0.078
History of CVA	82 (19.9%)	20 (17.9%)	62 (20.6%)	0.535
Hypertension	395 (95.6%)	107 (95.5%)	288 (95.7%)	0.949
Diabetes mellitus	243 (58.8%)	59 (52.7%)	184 (61.1%)	0.121
Dialysis parameters				
Kt/V (±SD)	1.64 ± 0.22	1.64 ± 0.2	1.63 ± 0.23	0.757
Water removal, kg (±SD) (pre-HD body weight minus post-HD body weight)	2.29 ± 0.91	2.31 ± 0.92	2.28 ± 0.91	0.758
Post-HD body weight, kg (±SD)	60.1 ± 14.1	62.5 ± 12.56	59.2 ± 14.59	0.035

Abbreviations: CAD, cardiac artery disease; CTR, cardiothoracic ratio; CVA, cerebrovascular accident; LVH, left ventricular hypertrophy; SD, standard deviation.

**Table 2:** Automatic segmentation and CTR calculation performance. mIoUs are calculated with the mask predicted by the neural network and the mask marked by the nephrologists; Dice coefficients are calculated with the mask predicted by the neural network and the mask marked by the nephrologists; R^2^ represents the correlations between the CTR predicted by the neural network and the CTR marked by the nephrologists with linear regression; relative changes represent the difference between the CTR predicted by the neural network and the CTR marked by the nephrologists.

Dataset	Training	Validation	Testing
Number of images	460 *	54 **	413 **
mIoU	0.943	0.935	0.950
Average Dice coefficient	0.970	0.966	0.974
R^2^	0.967	0.950	0.965
Relative change (difference between neural network mask and nephrologist-defined mask)	1.82%	2.0%	1.56%

Abbreviation: CTR, cardiothoracic ratio; mIoU, the mean of intersection over union; R^2^, the coefficient of determination. * JSRT dataset, 147; hemodialysis patients, 313. ** hemodialysis patients only.

**Table 3:** Comparison of CTRs calculated by nurse practitioners vs. nephrologists and those calculated by our neural network vs. nephrologists.

Method	Clinical Staff	Neural Network Model	*p* Value
Mean difference ± SD	1.52 ± 1.46%	0.83 ± 0.87%	<0.001
Absolute CTR bias ≥ 2% (*n*, %)	128 (31%)	35 (8%)	<0.001
R^2^	0.90	0.96	
Average time (second)	85	2	<0.001

Abbreviations: CTR, cardiothoracic ratio; SD, standard deviation. R^2^, the coefficient of determination.

## Data Availability

Detailed data is unavailable as a result of constraints imposed by privacy or ethical considerations.

## Supplementary Materials

The following supporting information can be downloaded at: https://www.mdpi.com/article/10.3390/diagnostics13081376/s1. Figure S1. (A) scatter plot comparing CTR by neural network with CTR by nephrologists. (B) Scatter plot comparing CTR by nurse practitioners with CTR by nephrologists. Figure S2. (A) Bland-Altman plot of CTRs by nurse practitioners and by nephrologists (B) Bland-Altman plot of CTRs by neural network and by nephrologists. Figure S3. Confusion matrix: 1.Left, Neural network vs. nephrologist: accuracy 94.92%, sensitivity 96.36%, specificity 90.99%. 2.Right, Clinical staff vs. nephrologist: accuracy 92.49%, sensitivity 90.07%, specificity 99.10%. Table S1 Comparison of Dice coefficient with the other articles.
