# Supplementary material for: Validation of an Automated Cardiothoracic Ratio Calculation for Hemodialysis Patients
Source: Diagnostics (Basel). 2023 Apr 9;13(8):1376. doi: 10.3390/diagnostics13081376 (PMC10137564; doi:10.3390/diagnostics13081376)
Supplement: Supplementary file 1 [file diagnostics-13-01376-s001.zip › diagnostics-2287792-supplementary.pdf]

diagnostics

MDPI

Article

Validation of an Automated Cardiothoracic Ratio Calculation for Hemodialysis Patients

Supplementary Materials:

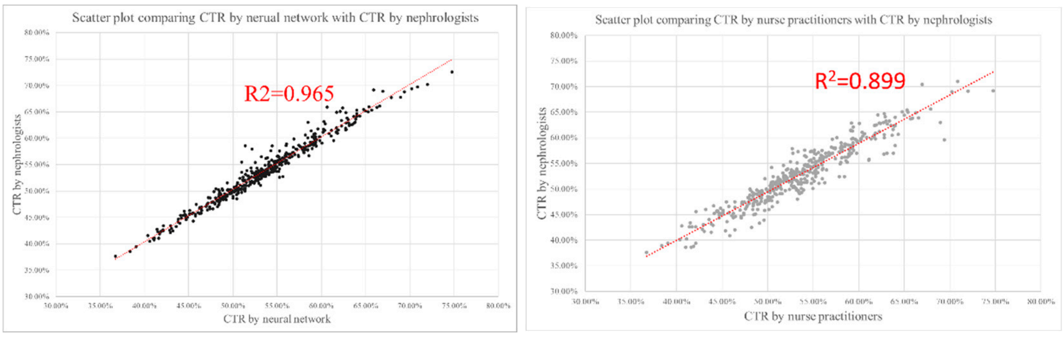

(A) (B)

Supplement Figure S1. (A) scatter plot comparing CTR by neural network with CTR by nephrologists. (B) Scatter plot comparing CTR by nurse practitioners with CTR by nephrologists.

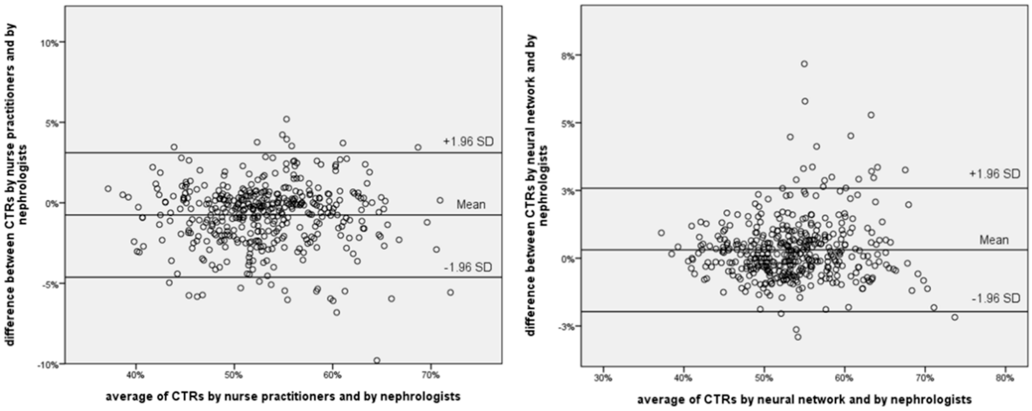

(A) (B)

Supplement Figure S2. (A) Bland-Altman plot of CTRs by nurse practitioners and by nephrologists (B) Bland-Altman plot of CTRs by neural network and by nephrologists

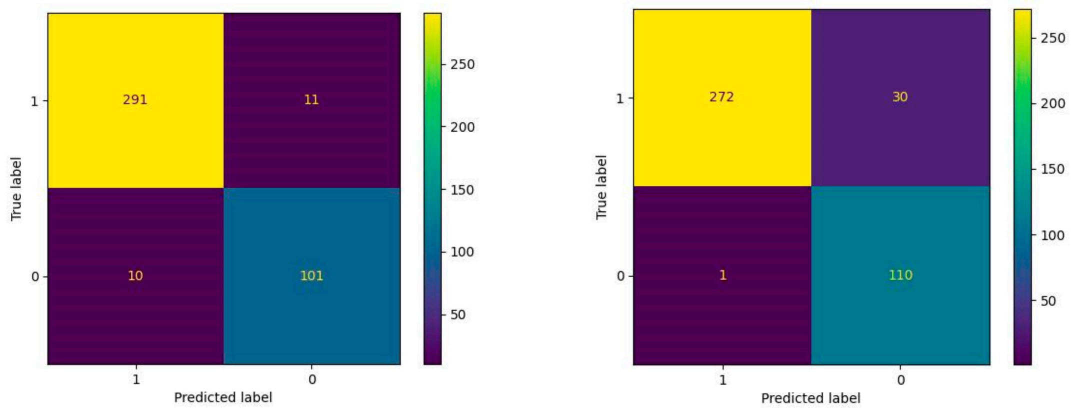

Neural network

Clinical staff

Supplemental Figure S3. Confusion matrix: 1.Left, Neural network vs. nephrologist: accuracy 94.92%, sensitivity 96.36%, specificity 90.99%. 2.Right, Clinical staff vs. nephrologist: accuracy 92.49%, sensitivity 90.07%, specificity 99.10%

Reference	Model	Dice coefficient	IoU	R^2
Chamveha et al.[1]	Unt (VGG-16)	0.97	N.A.	N.A.
Solovyev et al.[2]	Feature Pyramid Network (Resnet-50)	0.915	0.845	0.903
Eslami et al.[3]	Pix2pix	0.985	N.A.	N.A.
Our method	AlbuNet-34	0.974	0.95	0.965

Supplemental Table S1 Comparison of Dice coefficient with the other articles.
